# Ultrasonographic Findings in Periarthritis of the Shoulder and Their Correlation With Clinical Severity

**DOI:** 10.7759/cureus.108787

**Published:** 2026-05-13

**Authors:** Aditi Yadav, Raj Kumar Yadav, Osama Neyaz, Udit Chauhan, Varun Thomas Paul Panjikkaran, Karthik Sankar, Akash Rajkumar, Sandeep Dilip Ukey

**Affiliations:** 1 Physical Medicine and Rehabilitation, Vardhman Mahavir Medical College & Safdarjung Hospital, New Delhi, IND; 2 Physical Medicine and Rehabilitation, All India Institute of Medical Sciences, Rishikesh, Rishikesh, IND; 3 Physical Medicine and Rehabilitation, King George's Medical University, Lucknow, IND; 4 Radiodiagnosis, All India Institute of Medical Sciences, Rishikesh, Rishikesh, IND

**Keywords:** axillary recess, coracohumeral ligament, long head of biceps tendon effusion, periarthritis shoulder, rotator interval, ultrasound

## Abstract

Introduction: Periarthritis (PA) of the shoulder is a common cause of pain and disability. Although MRI is the standard imaging modality, ultrasonography (USG) is a cost-effective and accessible alternative. However, evidence correlating USG findings with clinical severity in Indian settings remains limited. This study evaluated the ultrasonographic findings in PA of the shoulders and determined their correlation with clinical parameters, including pain, disability, and passive range of motion (PROM).

Methods: This cross-sectional study included 79 patients with unilateral PA of the shoulder at a tertiary care academic institute in India. Pain was assessed using the Visual Analogue Scale (VAS), disability using the Shoulder Pain and Disability Index (SPADI), and PROM using a universal goniometer. Bilateral shoulder USG was performed to measure coracohumeral ligament (CHL) thickness, rotator interval (RI) thickness, axillary recess (AR) thickness, and long head of biceps tendon (LHBT) effusion. Statistical analysis included t-tests and correlation coefficients.

Results: USG parameters were significantly higher on the affected side compared to the unaffected side: CHL (2.73 ± 0.66 vs 2.09 ± 0.63 mm), RI (1.72 ± 0.53 vs 1.39 ± 0.35 mm), AR (3.85 ± 1.57 vs 2.30 ± 1.09 mm), and LHBT effusion ((1.40 (0.00-3.00) vs 0.00 (0.00-1.00) mm) (p < 0.001). PROM was significantly restricted in all movements. No significant correlation was observed between USG parameters and VAS or SPADI scores. However, AR thickness showed a fair positive correlation with PROM restriction (p < 0.05).

Conclusion: USG reliably detects structural changes in patients with shoulder pain and limited PROM in one clinically relevant plane. AR thickness correlates with functional limitation and may serve as a useful imaging marker.

## Introduction

Shoulder pain is considered one of the most common causes of musculoskeletal pain, preceded by neck and lower backache [1]. Periarthritis (PA) of the shoulder is one of the most prevalent causes of shoulder pain and disability in middle-aged populations, occurring in 2% to 4% [2], and is one of the most common causes of shoulder pain in India apart from fibromyalgia and cervical pathology [3]. PA of the shoulder, also known as adhesive capsulitis or frozen shoulder, is a gradual progression of movement restriction in all directions, with usual complaints of severe pain on the affected side [4].

With advances in microscopic and surgical technologies, it was discovered that structures such as the rotator interval (RI), the long head of the biceps tendon (LHBT), and the coracohumeral ligament (CHL) are associated with the pathogenesis of PA of the shoulder [5]. Also, increased fibroblasts and myofibroblasts, suggestive of a fibrotic process, have been reported in some studies [6]. Eventually, this suggests that the PA of the shoulder may begin as an immunological response, progress to inflammatory synovitis, and ultimately lead to capsule fibrosis [7]. The attachment of the capsule to the anatomical neck of the humerus is a source of pain and, when abruptly stretched, results in mechanical limitation of movement [8]. Primary or idiopathic PA of the shoulder presents with restricted shoulder movements, all of which are painful, with no underlying cause. Secondary PA occurs in association with diabetes, trauma causing muscle tears, previous shoulder surgery, prolonged immobilisation, thyroid disorders, Dupuytren's disease, and other autoimmune disorders [9-13].

Early stages of PA of the shoulder are often confused with other shoulder pathologies, which can be distinguished using MRI and ultrasonography (USG). MRI is considered the standard investigation for clinical correlation of PA of the shoulder, as it can identify joint capsule edema, thickness, and bicipital tendinitis very early in the course of pathology [14]. However, in the Indian context, its cost and availability are significant limitations. With the additional benefits of real-time dynamic examination and portability, USG is an inexpensive technology and may also be used as a diagnostic service in the community [15]. Very few studies show a comprehensive correlation between clinical presentation and USG findings in the PA of the shoulder. The primary objective of this study was to find out whether the values of USG parameters CHL thickness, AR thickness, RI thickness, and LHBT in PA of the shoulder are significantly different from the normal side, with secondary objectives being correlation between these USG parameters and clinical findings in PA, including pain (assessed using the Visual Analogue Scale (VAS)) [16], dysfunction (measured by the Shoulder Pain and Disability Index (SPADI)) [17], and passive range of motion (PROM) of the affected shoulder. The purpose of the present study was to evaluate the overall imaging findings and diagnostic utility of USG in PA of the shoulder rather than to compare subtypes separately. Therefore, primary and secondary cases were analyzed collectively and not as independent subgroups.

## Materials and methods

This cross-sectional study was conducted at the Department of Physical Medicine and Rehabilitation in All India Institute of Medical Sciences (AIIMS), a tertiary care teaching hospital in Rishikesh, India, over six months, from December 2022 to May 2023, with the approval of the Institutional Ethics Committee (approval number: AIIMS/IEC/22/601 dated 23/12/2022). All patients who attended the outpatient department during the study period with unilateral shoulder pain for at least one month and stiffness or PROM restriction (at least one clinically relevant plane) of the affected shoulder were included. Exclusion criteria encompassed patients with bilateral shoulder pain and surgery of the affected shoulder, muscle tears, bursitis, and osteoarthritis. Patients with a history of intra- or periarticular injection in the shoulder joint were excluded from the study. A total of 79 patients were enrolled in the study during the six months using a consecutive sampling method. Written informed consent was obtained from them, and their data were anonymized and kept confidential, in accordance with applicable institutional and governmental regulations governing ethical data use. They were examined for the assessment of pain using VAS [16], dysfunction using SPADI score [17], PROM of shoulders, and measurement of USG parameters, including CHL, AR thickness, RI thickness, and LHBT effusion thickness.

Clinical assessment

Patients rated their average shoulder pain on a 100 mm VAS (0-100), where 0 indicates no pain, and 100 indicates extreme pain [16]. All patients were examined for PROM in both shoulders. A deficit in PROM was noted in forward flexion (normal 0-180 degrees), IR (normal 0-90 degrees), ER (normal 0-90 degrees), and abduction (normal 0-180 degrees) using a universal goniometer in a sitting position. Post examination, patients completed the SPADI questionnaire [17].

USG parameters

A USG examination was performed by an experienced radiologist using a 7-13 MHz linear probe of the My Lab One USG device (Model 8100, Esaote, Genoa, Italy). It was performed in the normal and diseased shoulder of the same patient. The standard protocol was performed to scan the shoulder anatomy and exclude rotator cuff tears and bursitis.

CHL Thickness

**Figure 1:**
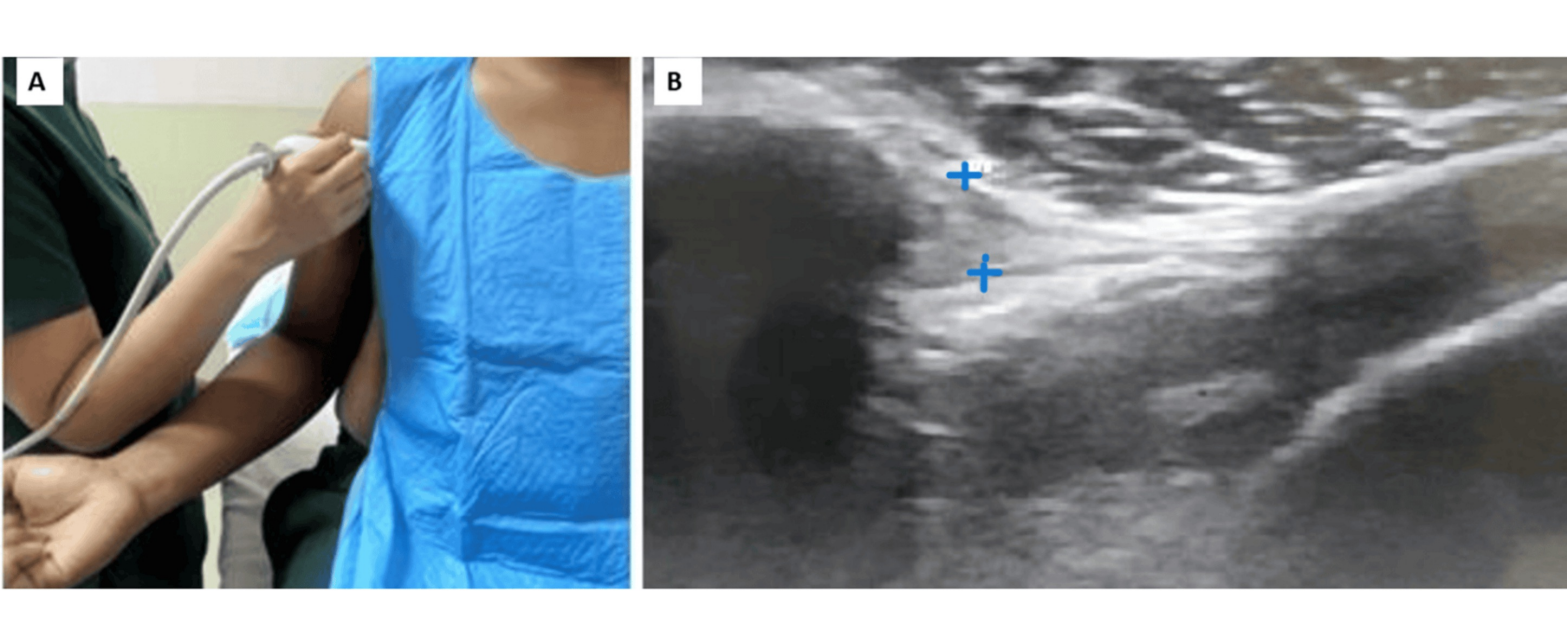
Measurement of the coracohumeral ligament thickness by ultrasonography A) Position of the probe; B) Ultrasound image

Examination was performed with the patient in a sitting position, with the humerus externally rotated in the available range to stretch out the CHL. The position of the transducer was on the lateral border of the coracoid process to get a longitudinal view of CHL. The CHL appears as a linear, hypoechoic band extending from the coracoid process to the RI in an inflammatory condition like PA, showing loss of fibrillar pattern [18]. We performed a dynamic examination to confirm that CHL was present with internal and external rotation. Longitudinal images of the CHL were obtained, and the CHL thickness slightly lateral to the coracoid process was assessed in bilateral shoulders (Figures 1A, 1B).

RI Thickness

**Figure 2:**
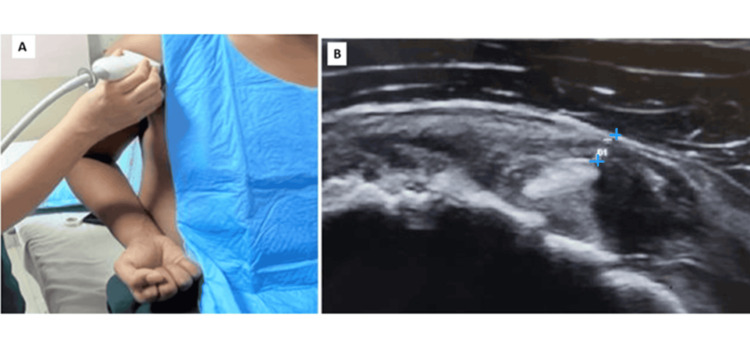
Measurement of the rotator interval thickness by ultrasonography A) Position of the probe; B) Ultrasound image

Examination was done with the patient sitting and the patient’s fist held at the side with the elbow flexed [19]. The shortest distance between the biceps long head tendon and peri-bursal fat, including the CHL, the superior glenohumeral ligament, and other RI tissues, was measured as RI thickness (Figures 2A, 2B).

AR Thickness

**Figure 3:**
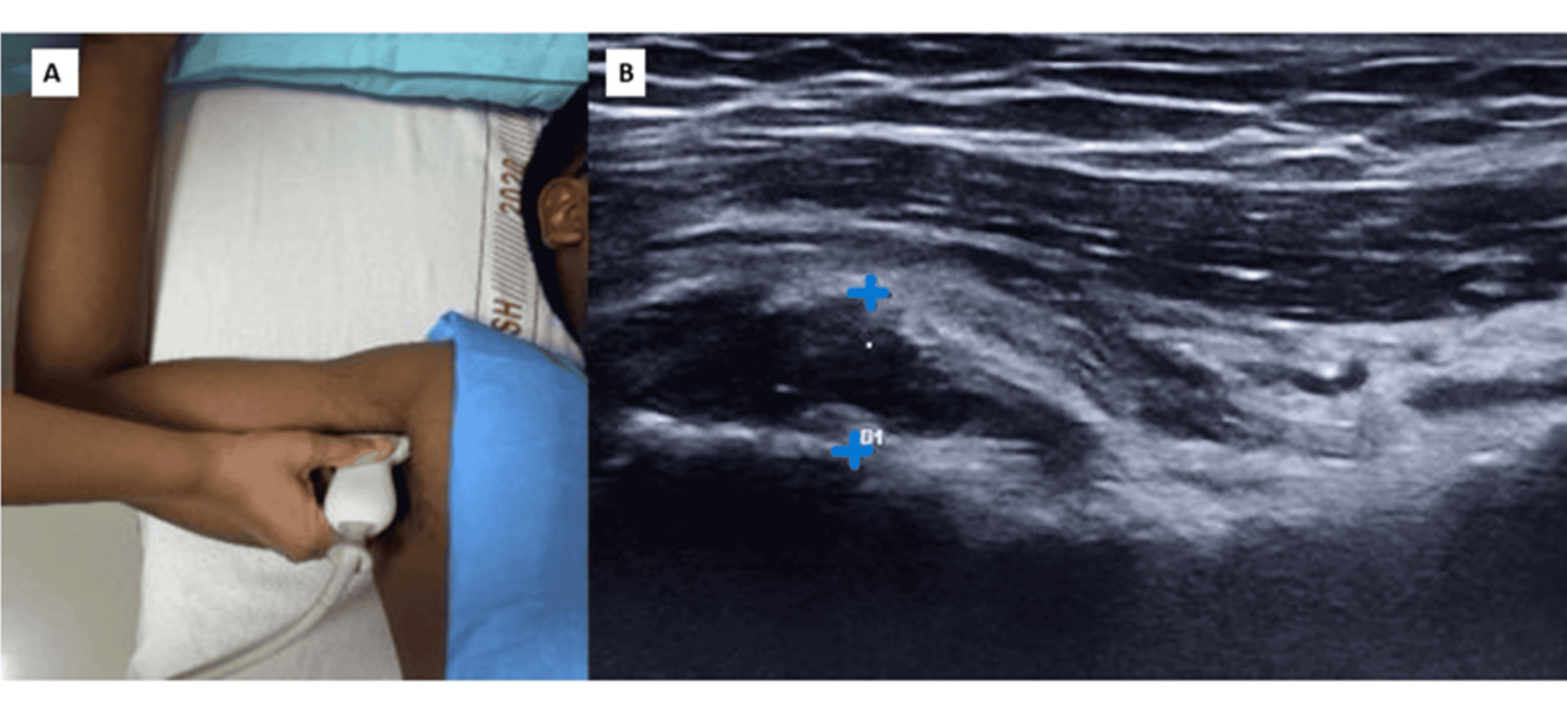
Measurement of axillary recess thickness by ultrasonography A) Position of the probe; B) Ultrasound image

The examination was done using the patient lying down, the shoulder abducted to 90 degrees or the maximal available range, the elbow bent at 90 degrees, and the forearm neutral. The USG probe was oriented in the longitudinal position on the midaxillary line in the long axis of the humeral shaft [20]. AR thickness was measured as the maximal distance between the bony cortex and the outer line of separation between the anterior and superficial joint capsule at the humeral surgical neck (Figures 3A, 3B).

LHBT Effusion Thickness

**Figure 4:**
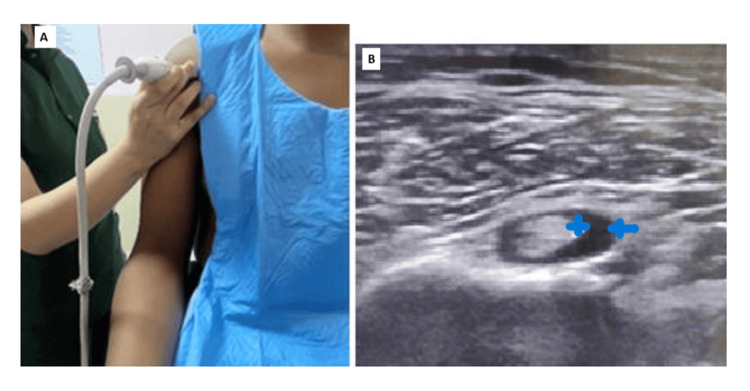
Measurement of long head of biceps tendon effusion by ultrasonography A) Position of the probe; B) Ultrasound image

The LHBT sheath effusion was scanned at the proximal humeral metaphysis level, with the patient sitting and the arm rotated internally. The probe was placed at the upper level of the bicipital groove to get the short-axis image of the biceps tendon. The maximal space between the points on the tendon sheath and the biceps tendon border was taken as the thickness (Figures 4A, 4B).

Statistical analysis

Descriptive statistics were used to characterize clinico-demographic variables. Data were checked for normal distribution using the Shapiro-Wilcoxon test. Continuous variables were reported as means and standard deviations for normally distributed data, and as medians for abnormally distributed data. Frequency count and percentage were presented for categorical variables. The student’s t-test was used to compare USG parameters between diseased and normal shoulders. A one-way ANOVA test was used to test more than two groups. Pearson and Spearman rank correlations were used to test the alliance between USG parameters and clinical variables. Every statistical test was two-sided and with 5% significance level.

## Results

A total of 79 patients were enrolled in the study. Detailed demographic and clinical data of all the patients were recorded. The mean age of the study group was 51.89 years. More than 70% of the study group was aged between 41 and 60 years, with a female preponderance (54%). More than half of the study group were unemployed at the time of recruitment, mainly homemakers, while one was a professional, two were semi-professionals, and eighteen were engaged in work that required arithmetic skills (according to the Revised Kuppuswamy Scale categorization, 2017) [21]. Almost half of the cases had the illness lasting less than four months, while nine cases lasted more than eight months. The mean duration of illness was 4.56 months; 53.2% had right-sided involvement, while 46.8% had left-sided involvement. The distribution of cases according to demographics and the presence of comorbidities is presented in Table 1.

**Table 1:** Socio-demographic variables of the study group and comorbidities

Socio-demographic variables	Number (Percentage; Total n = 79)
Age (years)	
31 – 40 years	6 (7.59%)
41 – 50 years	30 (37.97%)
51 – 60 years	26 (32.91%)
61 years and above	17 (21.51%)
Mean age in years ± SD	51.89 ± 8.87
Gender	
Male	36 (45.56%)
Female	43 (54.43%)
Occupation	
Unemployed	41 (51.89%)
Unskilled	5 (6.32%)
Semi-skilled	1 (1.26%)
Skilled	11 (13.92%)
Arithmetic skills	18 (22.78%)
Semi-professional	2 (2.53%)
Professional	1 (1.26%)
Comorbidities	
Diabetes mellitus (DM)	29 (36.7%)
Hypothyroidism	8 (10.12%)
Trauma	14 (17.72%)
DM+ hypothyroidism	2 (1.5%)

The mean VAS score was 60.9, and the SPADI pain score was 28.42, with a range of one to 48 out of 50. The SPADI disability mean score was 38.72 out of 80, with a range of 8-66 (Table 2).

**Table 2:** VAS Scores and SPADI scores of the study group VAS: Visual Analogue Scale; SPADI: Shoulder Pain and Disability Index

Clinical scales	Scores	Number (Percentage; Total n = 79)
VAS score (0-100)	Less than 50	17 (21.51%)
	50 – 70	38 (48.1%)
	More than 70	24 (30.37%)
	Mean VAS score ± SD	60.95 ± 20.21
SPADI total score	Mean	66.8
SPADI pain score	Mean ± SD	28.42 ± 10.73
	Range	1-48
SPADI disability score	Mean ± SD)	38.72 ± 14.85
	Range	8-66

The study compared the mean deficit in passive range of movement of the shoulder joint between the diseased and normal sides and found that the mean deficits in abduction, flexion, internal rotation, and external rotation were significantly greater on the affected side than on the unaffected side. The mean deficit in PROM of affected shoulder abduction movement was 46.2 degrees out of 180 degrees, for flexion 42.27 out of 180 degrees, 42.34 out of 90 degrees' deficit in internal rotation, and 39.74 degrees' deficit out of 90 degrees in external rotation movement (Table 3).

**Table 3:** Comparison of mean deficit of PROM and USG parameter measurements between affected/unaffected shoulders. t-test used for normally distributed data (T value); Wilcoxon signed rank test used for non-parametric data (Z value); Median (interquartile range used for non normally distributed data) USG: ultrasonography; LHBT: long head of biceps tendon; RI: rotator interval; CHL: coracohumeral ligament; AR: axillary recess; PROM: passive range of motion

Passive range of movement	Mean ± SD (deficit in degrees)		p-value	T score	USG measurements	Mean ± SD (thickness in mm)/Median (IQR)		p-value	Statistic
	Affected side	Unaffected side				Affected side	Unaffected side		
Abduction	46.2 ± 35.56	0.5 ± 3.54	<0.001	-4.21	LHBT	1.40 (0.00-3.00)	0.00 (0.00-1.00)	<0.001	Z=-8.12
Flexion	42.27 ± 32.57	0.75 ± 4.74	<0.001	-3.98	RI	1.72 ± 0.53	1.39 ± 0.35	<0.001	T=2.65
Internal rotation	42.34 ± 25.13	0.37 ± 2.5	<0.001	-3.45	CHL	2.73 ± 0.66	2.09 ± 0.63	<0.001	T=2.41
External rotation	39.74 ± 26.05	0.37 ± 1.92	<0.001	-3.72	AR	3.85 ± 1.57	2.3 ± 1.09	<0.001	T=2.12

In our study, the mean values of RI, CHL, and AR thicknesses (mm) on the affected side were 1.72 ± 0.53, 2.73 ± 0.66, and 3.85 ± 1.57, while on the normal side they were 1.39 ± 0.35, 2.09 ± 0.63, and 2.3 ± 1.09, respectively. The LHBT values showed a non-normal distribution and were therefore expressed as median and interquartile range (IQR). The median LHBT thickness in the affected shoulder was 1.40 (IQR: 0.00-3.00) mm, whereas in the unaffected shoulder it was 0.00 (IQR: 0.00-1.00) mm. All these values were statistically significant in the PA of the shoulder limb compared to the normal one (Table 3). Regarding the correlation between VAS, SPADI scores, and ultrasonographic measurements, none of the p-values were significant (Table 4). However, with ROM, we found a positive, fair, and statistically significant correlation between deficits in passive abduction, flexion, internal rotation, and external rotation on the affected side and the ultrasonographic measurement of the axillary recess (p-values = 0.022, 0.029, 0.023, and 0.018, respectively) (Table 4).

**Table 4:** Correlation of VAS score, SPADI score, and passive range of motion of the shoulder joint with USG measurements on the affected side Pearson and Spearman rank correlation test was applied; the p-value was significant at <0.05. VAS: Visual Analogue Scale; SPADI: Shoulder Pain and Disability Index; LHBT: long head of biceps tendon effusion; RI: rotator interval; CHL: coracohumeral ligament; AR: axillary recess; USG: ultrasonography

USG measurements	VAS score		SPADI pain score		SPADI disability score		Passive abduction		Passive flexion		Passive internal rotation		Passive external rotation	
	Correlation coefficient	p-value	Correlation coefficient	p-value	Correlation coefficient	p-value	Correlation coefficient	p-value	Correlation coefficient	p-value	Correlation coefficient	p-value	Correlation coefficient	p- value
LHBT	0.068	0.549	0.09	0.386	0.03	0.780	0.052	0.650	0.076	0.504	0.045	0.692	0.148	0.193
RI	-0.039	0.735	-0.21	0.052	-0.11	0.334	0.107	0.349	0.123	0.281	0.044	0.700	0.087	0.443
CHL	0.022	0.845	0.20	0.076	0.07	0.504	0.048	0.676	0.113	0.323	0.024	0.832	0.087	0.445
AR	-0.066	0.565	-0.01	0.920	0.19	0.087	0.258	0.022	0.246	0.029	0.256	0.023	0.266	0.018

## Discussion

This study evaluated different USG parameters in patients diagnosed with PA of the shoulder and their correlation with clinical parameters. This study included 79 subjects with PA of the shoulder. In our study, considering the general population distribution, the dominant right shoulder was affected more than the left. The dominant arm is more likely to be affected during acute inflammation, as it is used more in daily activities [22]. According to the study, PA of the shoulder is most prevalent in older persons (mean age 51.89 years), homemakers (51.89%), those with diabetes mellitus (37%), traumatic injury (17.72%), and women (54.43%). Increase in age, female gender, diabetes mellitus, traumatic injury, and involvement of the dominant side are well-known risk factors for PA of the shoulder [23,24]. Almost half of the cases had illness lasting less than four months, while nine cases had illness lasting more than eight months. The mean duration of illness was 4.56 months. This indicates that most patients were in the early stages of the pathology.

In our study, the ultrasound parameters LHBT, RI, CHL, and AR thicknesses were significantly higher on the affected side than on the unaffected side. CHL thickness in the affected shoulder had a mean value of 2.73 ± 0.66 mm compared to the mean value of 2.09 ± 0.63 mm in the normal shoulder. Many studies have shown evidence of CHL thickening on USG. Jong Geol Do et al. reported a CHL cutoff of 2.2 mm for diagnosing PA shoulder, with high sensitivity and specificity [25]. An existing study showed thickened CHL in 76% of PA shoulders, with an average CHL thickness of 3 mm in affected shoulders, and also concluded that USG is a reliable tool for measuring it [26]. Studies have indicated that CHLs are shortened and thickened in PA, limiting external rotation. Histological studies have demonstrated that this arises from fibroblastic proliferation in CHL. These histological changes are similar to those of Dupuytren’s superficial fibromatosis; thus, it may serve as a useful diagnostic parameter [6]. AR is located between the anterior and posterior bands of the inferior glenohumeral ligaments and is a part of the inferior glenohumeral joint capsule. Our study showed a mean AR thickness of 3.85 ± 1.57 mm on the diseased side, versus 2.3 ± 1.09 mm on the normal side, compared with other studies reporting mean AR thicknesses of 4.6 ± 1.6 mm and 2.6 ± 1.1 mm, respectively [27]. One of the key anatomical spaces in the shoulder is the RI, for which the anterior boundary is the anterior aspect of the supraspinatus tendon and the inferior boundary is the superior aspect of the subscapularis tendon. Our study showed an average RI thickness of 1.72 ± 0.53 mm on the affected side, compared with 1.39 ± 0.35 mm on the unaffected side. Neviaser AS demonstrated synovial proliferation and increased RI on arthrography in stage 2 PA [8]. Walmsley S et al. [28] and similar studies showed increased hypervascularity in RI during early stages. The LHBT arises from the glenohumeral joint capsule, and therefore, its effusion is associated with different shoulder joint pathologies. In our study, the median (IQR) effusion on the affected side was 1.40 (0.00-3.00) mm, which is comparable to the mean of 1.7 ± 1.6 mm from similar studies. In a retrospective study [29], the effusion within the biceps long head tendon sheath was observed in 58.42% of patients with PA of the shoulder. The average amount of the effusion within the LHBT sheath was 1.7 ± 1.6 mm.

Limitations

In our study, when evaluating the correlation between clinical values and ultrasonographic measurements, only AR thickening showed a statistically significant correlation across all ranges. No other USG parameters showed any significant correlation with VAS, SPADI, or PROM deficits. The reason for this could be that in PA, we usually have pain and disability more during the acute phase, while the changes in ultrasound parameters are mostly seen in the later stages of pathology. Also, being a cross-sectional study, a follow-up evaluation of the same patient in different stages of the pathologies was not done. This could have been more fruitful. More stringent inclusion and exclusion criteria may help achieve a more specific and homogeneous study population. The absence of blinding in the study may introduce observer bias. Since CHL is stretched and thinned out during normal external rotation, restricted external rotation might have affected the thickness of CHL. We also agree that the contralateral shoulder in patients with PA of the shoulder, particularly in diabetic individuals, may harbor subclinical inflammatory changes despite being clinically asymptomatic, which might affect baseline measurements.

## Conclusions

In conclusion, measuring CHL, AR, RI, and LHBT effusion thicknesses on USG can be utilized as a reliable tool for distinguishing normal from abnormal structures in the PA shoulder and ruling out other shoulder pathologies. Although the study found a positive correlation between AR thickness and PROM restriction, to examine the correlation between these USG parameters and clinical findings in the PA of the shoulder, we need a larger sample of patients with disease at different stages of the pathology.
